# Cross-site collaboration on infection prevention and control research—room for improvement? A 7-year comparative study in five European countries

**DOI:** 10.1186/s13756-022-01176-x

**Published:** 2022-11-03

**Authors:** Vanessa M. Eichel, Christina Brühwasser, Enrique Castro-Sánchez, Gabriel Birgand, Erik Bathoorn, Florian Salm, Nico T. Mutters

**Affiliations:** 1 Section for Hospital Hygiene and Environmental Health, Center for Infectious Diseases, Heidelberg University Hospital, Im Neuenheimer Feld 324, 69120 Heidelberg, Germany; 2 Infection Prevention and Hospital Hygiene, University Hospital Innsbruck, Anichstraße 35, 6020 Innsbruck, Austria; 3 Richard Wells Research Centre, University of West London, Brentford, Middlesex TW8 9GB England; 4 Regional Center for Infection Prevention and Control, Pays de La Loire, Nantes University Hospital, Nantes, France; 5 Department of Medical Microbiology, University of Groningen, University Medical Center Groningen, Groningen, The Netherlands; 6 Prevent Infect, Bettina-Von-Arnim-Str. 12, 79189 Bad Krozingen, Germany; 7 Institute for Hygiene and Public Health, University Hospital Bonn, Venusberg-Campus 1, 53127 Bonn, Germany

**Keywords:** Infection control, IPC, Collaboration, Cross-site, Europe

## Abstract

**Background:**

The spread of SARS-CoV-2, multidrug-resistant organisms and other healthcare-associated pathogens represents supra-regional challenges for infection prevention and control (IPC) specialists in every European country. To tackle these problems, cross-site research collaboration of IPC specialists is very important. This study assesses the extent and quality of national research collaborations of IPC departments of university hospitals located in Austria, England, France, Germany, and the Netherlands, identifies network gaps, and provides potential solutions.

**Methods:**

Joint publications of IPC heads of all university hospitals of the included countries between 1st of June 2013 until 31st of May 2020 were collected by Pubmed/Medline search. Further, two factors, the journal impact factor and the type/position of authorship, were used to calculate the Scientific Collaboration Impact (SCI) for all included sites; nationwide network analysis was performed.

**Results:**

In five European countries, 95 sites and 125 responsible leaders for IPC who had been in charge during the study period were identified. Some countries such as Austria have only limited national research cooperations, while the Netherlands has established a gapless network. Most effective collaborating university site of each country were Lille with an SCI of 1146, Rotterdam (408), Berlin (268), Sussex (204), and Vienna/Innsbruck (18).

**Discussion:**

The present study indicates major differences and room for improvement in IPC research collaborations within each country and underlines the potential and importance of collaborating in IPC.

**Supplementary Information:**

The online version contains supplementary material available at 10.1186/s13756-022-01176-x.

## Introduction

In every European country, infection prevention and control (IPC) specialists must tackle multiple challenges to maintain high standards for patient safety. The spread of SARS-CoV-2, multidrug-resistant organisms (MDROs), healthcare associated pathogens and the fight against healthcare-acquired infections requires evidence-based approaches. Furthermore, frequent patient transfers due to interdisciplinary need of treatment facilitate a possible spread of healthcare-associated pathogens and MDROs across wards, hospitals, regions and even borders [1–4]. Hospitals are usually part of regional transferal networks of the same region or country, and can therefore be challenged by the same outbreak strain, the same hygiene problem, or the same compliance issue. Communication and networking between IPC specialists and exchange of data, experiences and solutions between hospitals is therefore crucial, and cross-site collaboration in research among IPC specialists may play a key role for establishing and sustaining networks.

Building collaborations and extending networks across institutions, countries and nations is a course of action increasingly used to generate new knowledge [5, 6]. These synergies can lead to faster and more valid research results, high quality solutions, early adoption of methods, removing duplication of effort, and enrolling larger numbers of study participants [6]. Furthermore, scientific networking creates room for broader discussions and fosters higher chances of funding, helps to focus on research areas that are receiving less attention, and supports rapid improvement of patient safety [6].

However, the extent of scientific collaboration may vary from one country to another or between regions, with the influence of cross-site research collaborations in IPC on the output and quality of research not assessed yet. The aim of the present study was to analyse and compare the extent and quality of national research collaborations of IPC departments of all university hospitals located in Austria, England, France, Germany, and the Netherlands, as well as identifying network gaps and provide potential solutions.

## Methods

We performed a comparative study to evaluate the extent and quality of scientific collaborations between IPC heads over time in all national university hospitals in Austria, England, France, Germany, and the Netherlands. The study period was defined as 1st of June 2013 until 31st of May 2020. We measured all scientific publications of IPC heads (pub) and separately joint publications (jnt-pub) with other IPC heads. Additionally, data on scientific collaborations, data on the individual research performance i.e. H-indexes of the heads was also collected.

### Country inclusion

To investigate research collaboration regardless of country size, the goal was to include at least two small (Austria and the Netherlands) and two large countries (France and Germany) in Europe that are highly active in IPC research. In addition, England was included because it offers a unique and rather united health care system.

### Site identification

University hospitals of each country were included. In case of major cities with multiple university hospitals belonging to the same university (e.g. Paris) only the overall IPC head was included. Multiple heads were only included if there were multiple university hospitals belonging to different universities present (e.g. Amsterdam).

### Identification of the person in charge for IPC

Leaders of each IPC department of all university hospitals (sites) were evaluated by desktop-based research and/or by email/telephone requests to the institution.

Individuals were included if they were holding the academic head position of the IPC department or service at some point during the study period. If the IPC head at a site had changed during the study period, then all IPC heads of this site were included in the analysis. Sites were excluded if the IPC head identified had not published any Pubmed/Medline-indexed research during the study period.

### Profile of authors

The publications of each identified author were evaluated by Pubmed/Medline search using the last name and several variations of the first name (full first name, initials, initials and city name of site of the University Hospital). Additionally, each detected publication was screened manually to verify the authorship. The filter tool was set to the period each author was holding the position of leader of IPC at the university hospital during the study period. Pubmed was chosen as a data search tool due to its status as a broad used reference source in the biomedical-, life-, and health science community, and its free access to a broad population.

Furthermore, the Hirsch-Index (H-Index) for all authors was identified at the same date, 21st of June 2021, using the Web of Science database. To calculate the mean H-Index for a site, the H-Index of each author from the site was multiplied by the years of holding the leader position (together maximum 7 years) and divided by 7.

### Joint authorship network

To measure the grade of professional networks of scientists, the well-documented record of joint authorship provides a helpful evaluation tool [7]. Evaluation of joint authorship records was conducted using Pubmed/Medline search. Each author was paired with all other authors of the same country by adding an "AND" between the names (last name, first letter of first name) in the search function.

A joint authorship was defined as the joint authorship of peer reviewed research papers (articles, reviews and letters) by a minimum of two authors of the same country. Only outputs published during the time both authors were leads of IPC were included. Documents were excluded if authors were not listed individually but as part of a consortium or research group. In addition, books and conference papers were excluded.

### Network analysis

To evaluate the impact of the scientific collaboration and the contribution of a site, the joint publications were weighed by two more factors, the journal impact factor and the type of authorship position. Therefore, each publication was multiplied by factor 5 for a first or last authorship, resulting in authorship points (APs) for the publications.

The APs were then multiplied by the most recent 5-year impact factor (IF) of the journal. To avoid multiplications by zero where a journal may have not received an IF yet, one point was added to the IF (IF + 1) before the multiplication. Impact factor search was conducted via the Clarivate Journal Citation Report and Scimago.

Finally, the sum of all weighted joint publications of a site was calculated and named the Scientific Collaboration Impact (SCI) of the site.

I.e.: ***SCI (X)*** = ***∑ AP x (IF + 1).***

### Network visualization

To visualize the nationwide collaboration networks, Cytoscape open-source software 3.8.2. was used.

Nodes indicate each site of a country. The intensity of the colour of a node defines the strength of the total SCI. The weighted collaborations between one site and a collaborating site of each year are indicated as width of edges in a different greyscale colour mapping.

### Statistical analyses

To evaluate correlations between (mean) joint publications and (mean) publications, Pearson correlations coefficient (r) and *p-values* were calculated. The significance threshold was set at 0.001. R-values: > 0.7 = strong correlation; 0.5–0.7 = moderate correlation, > 0.4 = weak or no correlation; p < 0.05 = significant, p-value threshold: 0.001.

## Results

**Fig. 1:**
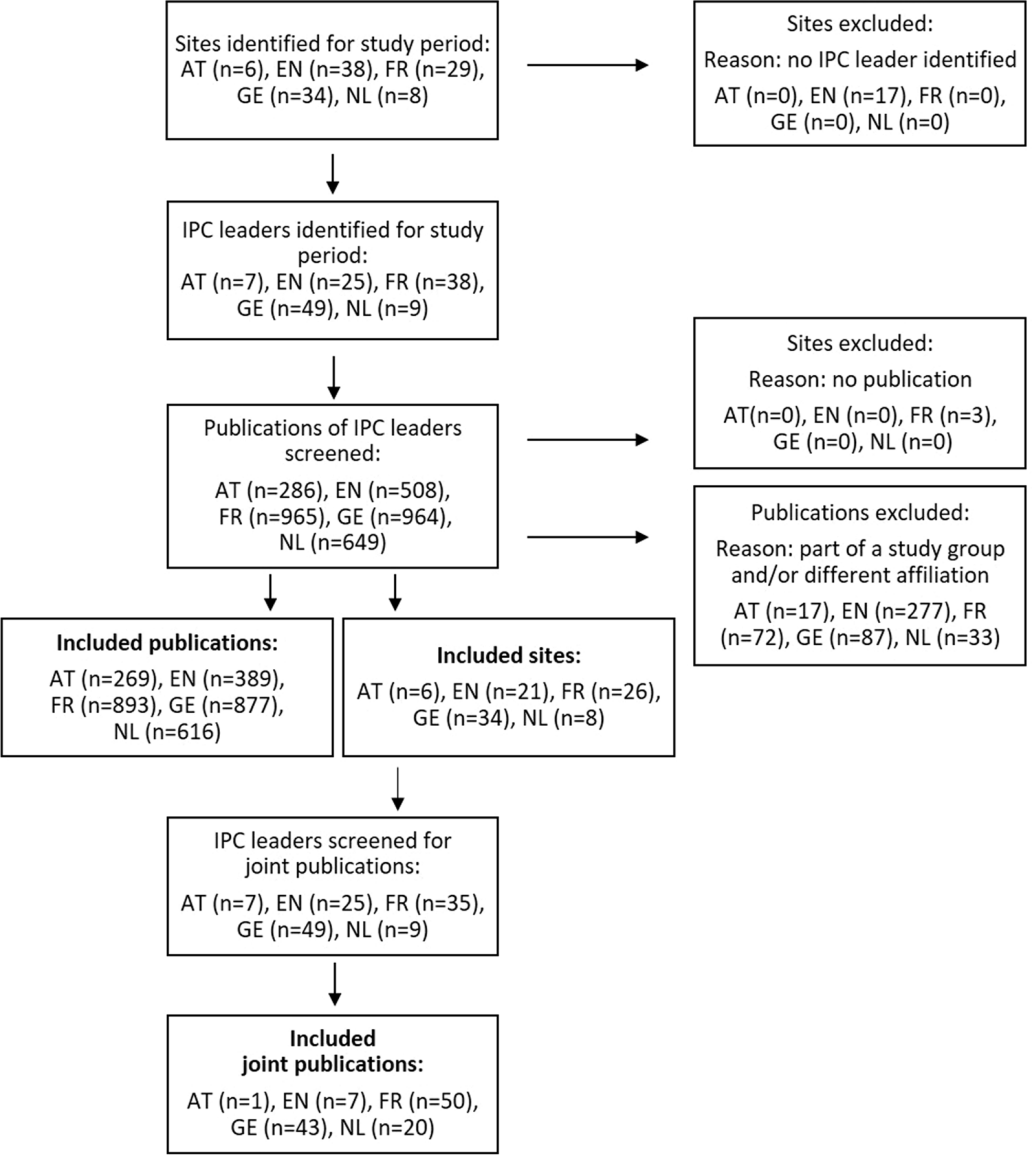
Flow chart of site, IPC head, and publication identification. Identification of university hospitals of five European countries and the person in charge of ICP at the site for the study period and following identification of publications within each country. *AT* Austria, *EN* England, *FR* France, *GE* Germany, *NL* The Netherlands, *IPC* Infection Prevention and Control

In five European countries, 95 sites were identified (Additional file 1: Table S1). At these sites, 125 leaders responsible for IPC who had been in charge during the study period were identified (see Fig. 1).

### IPC research collaboration between countries

The evaluation of scientific collaboration in IPC of each country yielded interesting differences (Table 1). Collaboration was established in 100% (8/8) of sites in the Netherlands. In Germany, collaborating work was performed by 76% (26/34). In France 62% (16/26) of sites had at least one national collaborating site, while only 33% (2/6) of sites in Austria collaborated, and 29% (6/21) in England.

**Table 1:** Collected data of five European countries including sites, Scientific Collaboration Impact, publications, and H-Index

	Sites	Mean SCI/site	Mean jnt-pub/site	Mean pub/site	jnt-pub/pub [%]	Mean H-Index/site
Austria	6	6	0.17	45	0.4	23
The Netherlands	8	186	2.50	77	3.2	35
England	21	21	0.33	19	1.8	16
France	26	201	1.92	34	5.6	20
Germany	34	65	1.26	26	4.8	22

*SCI* Scientific Collaboration Impact, *pub* publications, *jnt-pub* joint publications between two or more sites within the country

Slightly different, the mean SCI score per site was highest in the French network (201), followed by the Netherlands (186), and Germany (65), while again England (21) or Austria (6) had lower scores. It needs to be considered, however, that high SCI scores indicate high impact of collaboration, regardless in which way that has been achieved, e.g. multiple collaborating sites, repeating collaboration between two sites, or publications in high impact journals. Even though Germany had a higher network extent (76% vs. 62%), the mean SCI scores (65 vs. 201) and total numbers of collaborations were lower than in France (61 vs. 78) due to publications in journals with minor impact. Austria had only one collaboration between two sites during the study period. This led to a comparable percentage of collaborating sites (33%) to England (29%) but to a lower mean SCI score (6 vs. 21). Furthermore, also the mean number of joint publications per site was highest in the Netherlands (2.50), followed by France (1.92), and Germany (1.26).

### Research collaboration on ICP within included countries

**Fig. 2:**
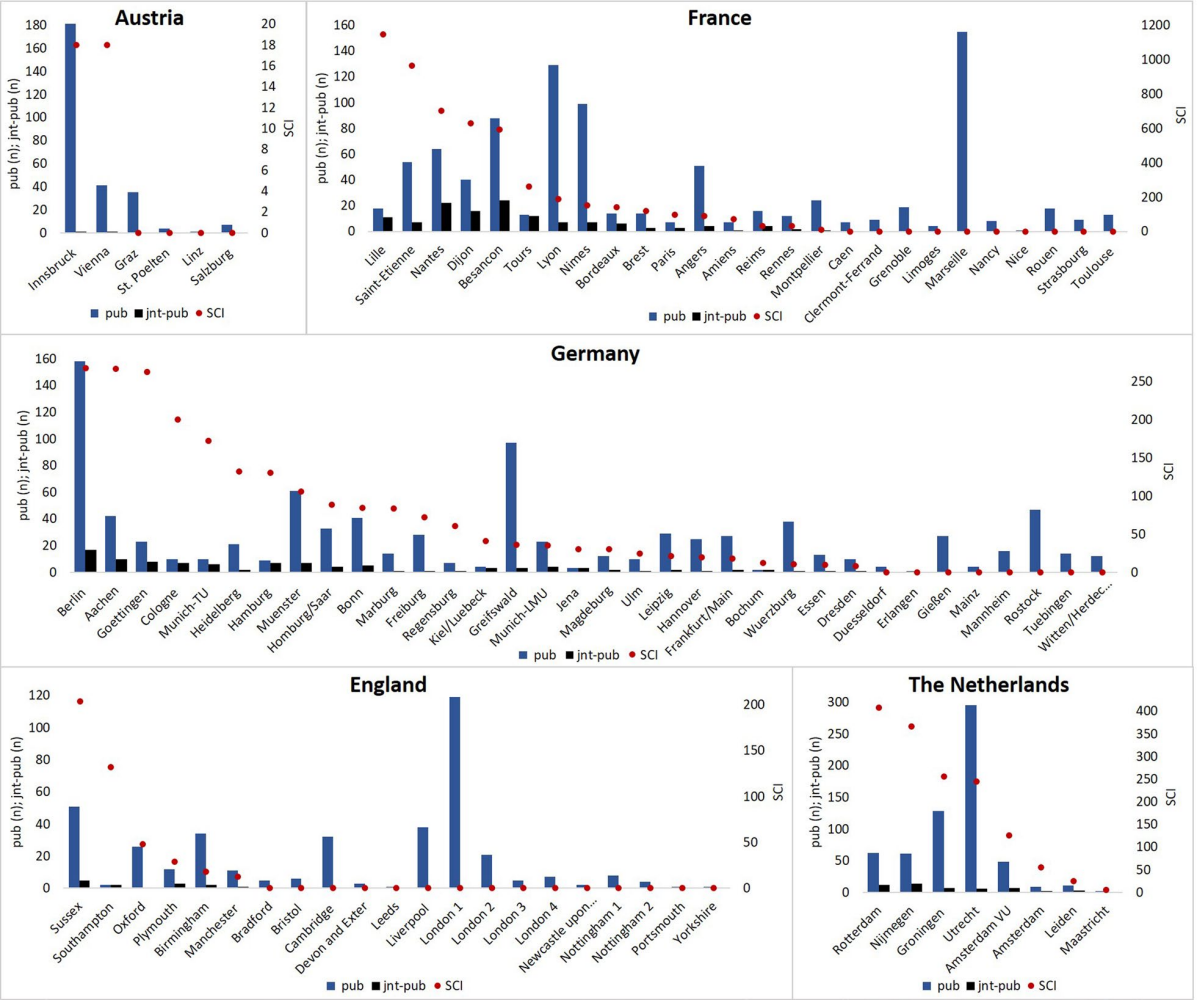
Scientific Collaboration Index (SCI), all publications (pub), and joint publications (jnt-pub) per site of each country

The mean SCI per site varied between 6 (Austria) with only one published scientific collaboration, and 201 (France) with 50 joint publications. Taking a closer look within the countries, with a score of 18 each, Innsbruck and Vienna equally had the highest SCI of Austria. Sussex (204, England), Lille (1146, France), Berlin (268, Germany), and Rotterdam (408, the Netherlands) were identified as sites with the highest SCI of their countries. Determinations of mean H-Indexes resulted in a range between 16 (England) up to 35 (the Netherlands). SCI, publications without cross-site collaboration and joint publications per site of each country are prented in Fig. 2.

**Fig. 3:**
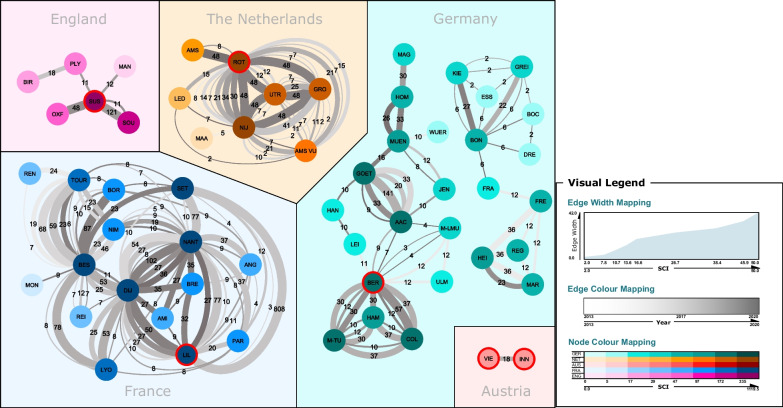
IPC Network analyses of University hospitals of five European countries. Red circles mark sites with highest SCI of a country. Nodes: sites of a country; Edges: SCI between a site and it’s collaborating site; Node colours mapping: strength of SCI; Edges width mapping: SCI; Edges grey-colour mapping: year of SCI; Numbers on edges: SCI of the year. Sites without connections are not depicted

To evaluate the scientific collaborations in IPC over time, the national networks were visualized in Fig. 3.

## Discussion

In the present study, research collaboration networks of IPC departments at all university hospitals located in Austria, England, France, Germany, and the Netherlands were analysed and compared for a period of seven years. All five countries and their 95 included university hospitals were evaluated for persons in charge for IPC during the study period. Network diagrams of 121 joint publications, weighted by type of authorship and impact, were used to visualize differences in scientific collaboration between the sites and counties.

The network visualization delivered some remarkable results. While some countries, such as Austria, have only very limited national research cooperations, others, such as the Netherlands, have established a gapless network including all university hospitals in the country. Of interest is also the comparison between capitals. While Berlin is the most connected site in Germany, Paris and Amsterdam are not the best nationally connected of their country, Vienna has only one joint published project, and London did not contribute to national joint publications.

There is, however, a peculiarity affecting the data on Paris. In Paris many local university hospitals do have their own IPC team. For example, in the case of the Public Hospitals Assistance of Paris, thirty-nine hospitals stand under the same administrative umbrella, having their own IPC team, all coordinated centrally. Some heads of IPC teams in the 39 sites hold professorships and are scientifically speaking very active. However, they were not included in our study since they did not meet our inclusion criteria. The national level connectedness of Paris is therefore underestimated in our study.

The comparison of national networks could be affected by the different health care systems, research funding schemes, sizes, and number of hospitals. Therefore, to compare output and impact of national networks within the countries, extension of networks and numbers of joint publications are discussed for every country in the following.

### Distinct IPC research collaborations within each country

Austria showed a rather high rate of mean publications (n = 45) and mean H-Indexes (23) per site, indicating strong internal research activities, but only one national collaboration. The research was driven by a single strong site, Innsbruck, with 181 publications and a H-Index of 62. Other sites did not publish many publications, ranging from 41 to one, or H-Indexes (26 to 2), suggesting a lower focus on IPC research at university level. Compared to the other four investigated countries, the national IPC university network in Austria has room to grow with only one single collaboration between Vienna and Innsbruck. The low proportion of joint publications to the total amount of publications (1 vs. 269) at all sites offers broad room for improvement. It would be plausible to assume that sites with strong research activities enhance collaborations. However, the national collaboration level did not show any relation to the overall research activity in Innsbruck and Vienna. Even though heads of departments and IPC leaders might be connected in a professional way by and to national IPC and hygiene societies, the national research collaboration of university hospitals in IPC appeared to be relatively weak compared to the other evaluated countries. Since the specialization in IPC is done together with Clinical Microbiology, there could be less sense of community and difficulties in networking due to a larger number of graduates. There is broad national scientific collaboration in the development of IPC guidelines through the Austrian Society for Hygiene, Microbiology and Preventive Medicine (ÖGHMP). However, obviously does this not lead to extensive national research cooperations. Also, the Austrian Society specifically for Hospital Hygiene (ÖGKH) focuses mainly on professional policy issues and is hardly involved in scientific research. Still, IPC experts and leaders may already be united in both societies, so they could function as advocates for more national and cross-site collaboration in IPC research.

The Netherlands showed highly active research with high-impact research outputs, as indicated by the high mean number mean publications (n = 77) and mean H-Indexes (35) per site. Additionally, the mean number of national collaborations per site was high (n = 5.25), and each site was involved in an average of 2.5 joint publications. Although, when comparing the proportion of mean joint publications to mean overall publications (0.03), the Netherlands could also increase the proportion of national co-authored publications. Especially Amsterdam VU, Groningen and Utrecht with the lowest joint publications compared to the total number of publications (n = 7 vs. 48; n = 7 vs. 128; n = 6 vs. 295) in the country. Still, the Netherlands represent the strongest national scientific IPC network in Europe producing high impact publications in plenty of collaborations. A possible explanation for this may be that in the Netherlands, there is a widespread awareness of the strengths of collaborative network approaches in infection control, as reflected by frequent activities in European study groups and cross-border programs. This network approach is also mentioned as an important strategy to combat the spread of highly resistant microorganisms on a regional level in a vision document from the Dutch Society of Medical Microbiology (NVMM). On top of that, nationwide networking begins early during IPC specialization, because nationwide joint courses are a mandatory part of the well-structured IPC specialization program for physicians.

England had rather low IPC research activities, measured by mean publication rate (19) and mean H-Index per site (16). The rate of mean joint publications (0.33) was low in proportion to the mean rate of publications (19). Southampton, however, showed a one-to-one proportion of joint publications to publications in combination with a high SCI (264), indicating a strong focus on national collaborating work compared to the other sites in England. However, it is difficult to draw conclusions about the IPC community and their research in England. Due to a different system in terms of the status and even definition of university hospital (i.e., ‘NHS trusts’ instead of direct university hospital) and the relations between the healthcare and educational system, it is rather complicated to compare England with the other four countries, with some existing collaborations potentially missed, and difficulty to assign IPC heads to certain hospitals with the ease demonstrated in other countries, as some organisations have ceased to exit following mergers with others or due to national reorganisations.

A high mean SCI per site of France (201) indicates a strong national IPC network. Further, France offered the highest number of national co-authored publications (n = 50) compared to the other four investigated countries. However, a low mean H-Index (20) and low mean numbers of publications (n = 34) might imply less research activity in IPC overall. Nevertheless, the high mean SCI per site suggests that the impact of national collaborations is rather high. Hence, the network focusses on the production of few high-impact factor publications instead of low-impact mass production. The French sites Bordeaux, Dijon, Lyon, Paris, and Tours have a strong focus on collaborating work, reading their SCI and proportions of joint publications to total publications in seven years. Amongst all five countries, France holds a highly active national network in IPC.

Germany presents an overall moderate research activity with a mean H-Index scores per site (22) and mean publications per site (n = 26). Further, a mean of 1.79 collaborations and 1.26 joint publications per site in seven years could be an incentive to improve and activate national collaborating work. However, the portion of joint publications among the overall publications was high, suggesting that when IPC research is conducted, it typically does involve national collaboration. Although Aachen, Berlin and Heidelberg did have high SCI scores (267; 268; 132) and a high amount of publications (n = 42; n = 158; n = 21), however the joint publication rate (0.24; 0.11; 0.10) was low, indicating few activities in national networking, but with good impact. Hamburg, Cologne and Munich TU showed the best collaborating indices, when combining SCI and the quotient (130; 201; 173 and 0.78; 0.70; 0.60). Of interest is that the Germany network in the mean has an extent of 76%, however, the visualization of the network shows that in Germany are basically three independent networks in place. These three networks have almost none or no connections to the other networks and are in this very different to the other European networks. Hence, connecting these independent networks with each other might increase the effect on research significantly and perhaps could result in scores comparable to France or the Netherlands, since lots of potential might be unused at the moment.

Interestingly, in every country at least one university hospital exist that does not have many national collaborations, but one of the highest number of overall publications in the country, i.e. the university hospitals of Innsbruck, Marseille, Utrecht, Greifswald, and Imperial College London (see Fig. 2). Hence, these university hospitals might be able to easily increase the nations network connectedness by focussing more on national collaborations since they are already having one of the most active IPC research heads.

### Advantages of research collaborations in IPC

To improve IPC research with multicentre collaborations has already been discussed in the past [7]. However, big collaborations can be conflict ridden by disagreements in leadership, methodology, or writing style [8] and also are influenced by general cultural aspects (Hofstede model). Additionally, they can be time-consuming for the involved IPC specialists, considering the limited time for research [7]. Nevertheless, linked advantages might be stronger research networks, better research outcomes and higher chances of funding and usually overrule disadvantages [6, 7]. External validity of results is higher when resulting from multi-centre trials compared to single-centre studies. They could gain far-reaching attention by the IPC community and new research partners for a site or country, but equally for a single researcher. The chance of publishing in a high impact journal might increase by establishing one or multiple (inter)national collaborations [9]. High impact factor publications could also be found when collaborating in the present study, as France and the Netherlands have achieved one or more publications in high impact factor journals such as the Lancet Infectious Diseases or the New England Journal of Medicine, during the investigated period. Strong researchers often do have an increased attention of the community by publishing in international journals and with international collaborations. Publishing in well-read journals might be of advantage in reaching a bigger IPC community and raising awareness for a specific topic. Furthermore, collaborating sites might benefit from knowledge and data exchange, could discuss important national IPC issues and develop solutions together, leading to improvement and standardization of national IPC guidelines.

### Individual benefits for the collaboration sites

Successful cross-site research collaborations might also imply a benefit for the individual research reputation of the involved sites. One of our hypotheses was that a high number of collaborations would also result in high quantity of citations of the sites researchers. However, this could not be verified, as there was no correlation between the number of collaborations per site and the mean H-Index of the site. As a limitation, the H-Index indicates the entire carrier of scientist, and cannot easily be adjusted to only a period of time. Therefore, it is possible that the H-Index resulted from research before an author was affiliated as head of IPC with a site.

Still, the national research collaborations that resulted in high impact joint publications contribute to the H-Index of a site. Further, sites with more collaborating publications were positively correlated with higher total numbers of publications, indicating ambitious researches for these sites.

The present study bears some limitations. Due to availability of data, only the leaders of IPC departments were included for joint publication search, that could have led to missing collaborations if the IPC lead was not listed as author of a research article. However, to minimize data gaps and biases, and to equalize chances for all countries and sites, the search was focused on IPC heads as representatives of each site. Additionally, the diversity in healthcare-systems of the investigated countries could have influenced the identification of university hospitals and IPC leads.

Nevertheless, this study indicates differences and room for improvement in IPC research collaborations within each country and underlines the potential and importance of collaborating in IPC.

To promote cross-site collaboration in IPC research we would suggest the following: Firstly, we propose the identification of hubs and gaps in the network and the creation of benchmarks in terms of targets to improve connectiveness. For example, the target to connect non-associated facilities with at least one project next year, and subsequently the appointment of persons in charge in the concerned facilities. The results of our study could be used as blue print.. In particular, sites with a strong research activity but few collaborations such as the university hospitals of Innsbruck, Marseille, Utrecht, Greifswald, and Imperial College London might easily raise their collaboration rate. Secondly, networking could be improved as a soft-skill development training. For instance, starting national networking at an early stage, e.g. during the IPC specialization, by organizing national exchange events and meetings for young doctors in training might act as seed event which could blossom in long-term scientific exchange. This could be supported by national grants for young doctors and PhDs issued by the national scientific societies. These grants could define as a condition that a minimum of 2 young researchers from different facilities need to be involved. Lastly, we must create awareness of the importance and advantages of national and international cohesion in this essential topic and support cooperative tendencies rather than competitive tendencies.

## Conclusions

The network visualizations presented here might provide insights and connecting points for national researchers to establish new collaborations, as cross-site, comprehensive IPC research is desirable for every country in the fight against the spread of infectious pathogens.

## Supplementary Information

**Additional file 1**. **Table S1:** Included university hospitals, Scientific Collaboration Impact, publications, and H-Index.

## Data Availability

The datasets used and analysed during the current study are available from the corresponding author on reasonable request.

## Abbreviations

IF: Impact factor
IPC: Infection prevention and control
jnt-pub: Joint publications
MDRO: Multidrug-resistant organisms
Pub: Publications
SCI: Scientific Collaboration Impact
