# Supplementary material for: Cross-site collaboration on infection prevention and control research—room for improvement? A 7-year comparative study in five European countries
Source: Antimicrob Resist Infect Control. 2022 Nov 3;11:131. doi: 10.1186/s13756-022-01176-x (PMC9631580; doi:10.1186/s13756-022-01176-x)
Supplement: Supplementary file 1 — Additional file 1. Table S1: Included university hospitals, Scientific Collaboration Impact, publications, and H-Index. [file 13756_2022_1176_MOESM1_ESM.docx]

**Supplement**

**Table S1:** Included university hospitals, Scientific Collaboration Impact, publications, and H-Index.

| COuntry | Site | Abbreviation | UNiversity Hospital | Mean SCI | Mean pub | Mean  jnt-pub | jnt-pub /  pub [%] | Mean H-Index |
| --- | --- | --- | --- | --- | --- | --- | --- | --- |
| The Netherlands | Amsterdam | AMS | University Medical Centers Amsterdam | 56 | 9 | 2 | 0.22 | 14 |
|  | Amsterdam VU | AMS VU | VU University Medical Center Amsterdam | 126 | 48 | 7 | 0.15 | 42 |
|  | Groningen | GRO | University Medical Centers Groningen | 255 | 128 | 7 | 0.05 | 52 |
|  | Leiden | LED | Maastricht University Medical Center | 25 | 11 | 3 | 0.27 | 10 |
|  | Maastricht | MAA | Leiden University Medical Center | 5 | 2 | 1 | 0.50 | 2 |
|  | Nijmegen | NIJ | Radboud University Medical Center | 367 | 61 | 14 | 0.23 | 49 |
|  | Rotterdam | ROT | Erasmus University Medical Center | 408 | 62 | 12 | 0.19 | 32 |
|  | Utrecht | UTR | University Medical Centers Utrecht | 245 | 295 | 6 | 0.02 | 82 |
| Germany | Aachen | AAC | University Hospital - RWTH Aachen | 267 | 42 | 10 | 0.24 | 22 |
|  | Berlin | BER | Charité University Hospital Berlin | 268 | 158 | 17 | 0.11 | 50 |
|  | Bochum | BOC | Ruhr-University Hospital Bochum | 12 | 2 | 2 | 1.00 | 13 |
|  | Bonn | BON | University Hospital of Bonn | 85 | 41 | 5 | 0.12 | 28 |
|  | Dresden | DRE | University Hospital of Dresden | 8 | 10 | 1 | 0.10 | 11 |
|  | Duesseldorf | DUE | University Hospital of Düsseldorf | 0 | 4 | 0 | 0.00 | 6 |
|  | Erlangen | ERL | University Hospital of Erlangen | 0 | 1 | 0 | 0.00 | 4 |
|  | Essen | ESS | University Hospital of Essen | 10 | 13 | 1 | 0.08 | 10 |
|  | Frankfurt/Main | FRA | University Hospital Frankfurt am Main | 18 | 27 | 2 | 0.07 | 16 |
|  | Freiburg | FRE | University Hospital Freiburg | 73 | 28 | 1 | 0.04 | 40 |
|  | Gießen | GIE | University Hospital of Gießen/Marburg | 0 | 27 | 0 | 0.00 | 15 |
|  | Goettingen | GOET | University Hospital of Göttingen | 263 | 23 | 8 | 0.35 | 16 |
|  | Greifswald | GREI | University Hospital of Greifswald | 36 | 97 | 3 | 0.03 | 39 |
|  | Hamburg | HAM | University Hospital of Hamburg-Eppendorf | 130 | 9 | 7 | 0.78 | 23 |
|  | Hannover | HAN | University Hospital of Hannover | 20 | 25 | 1 | 0.04 | 28 |
|  | Heidelberg | HEI | Hospital of Medical University of Heidelberg | 132 | 21 | 2 | 0.10 | 22 |
|  | Homburg/Saar | HOM | University Hospital of Saarland | 89 | 33 | 4 | 0.12 | 31 |
|  | Jena | JEN | University Hospital of Jena | 30 | 3 | 3 | 1.00 | 9 |
|  | Kiel/Luebeck | KIE | University Hospital Schleswig-Holstein | 41 | 4 | 3 | 0.75 | 7 |
|  | Cologne | COL | University Hospital of Cologne | 201 | 10 | 7 | 0.70 | 10 |
|  | Leipzig | LEI | University Hospital of Leipzig | 21 | 29 | 2 | 0.07 | 25 |
|  | Magdeburg | MAG | University Hospital of Magdeburg | 30 | 12 | 2 | 0.17 | 25 |
|  | Mainz | MAI | University Hospital of Mainz | 0 | 4 | 0 | 0.00 | 9 |
|  | Mannheim | MANN | University Hospital of Mannheim | 0 | 16 | 0 | 0.00 | 3 |
|  | Marburg | MAR | University Hospital of Gießen/Marburg | 84 | 14 | 1 | 0.07 | 22 |
|  | Munich-LMU | M-LMU | Ludwig-Maximilian University Hospital Munich | 35 | 23 | 4 | 0.17 | 11 |
|  | Munich-TU | M-TU | Technical University Hospital Munich-Rechts der Isar | 173 | 10 | 6 | 0.60 | 9 |
|  | Muenster | MUEN | University Hospital of Münster | 106 | 61 | 7 | 0.11 | 76 |
|  | Regensburg | REG | University Hospital of Regensburg | 60 | 7 | 1 | 0.14 | 30 |
|  | Rostock | ROS | University Hospital of Rostock | 0 | 47 | 0 | 0.00 | 44 |
|  | Tuebingen | TUEB | University Hospital of Tübingen | 0 | 14 | 0 | 0.00 | 16 |
|  | Ulm | ULM | University Hospital of Ulm | 24 | 10 | 1 | 0.10 | 27 |
|  | Witten/Herdecke | WIT | University Hospital Witten/Herdecke, Köln-Merheim | 0 | 12 | 0 | 0.00 | 21 |
|  | Wuerzburg | WUER | University Hospital of Würzburg | 10 | 38 | 1 | 0.03 | 47 |
| Austria | Graz | GRA | University Hospital of Graz | 0 | 35 | 0 | 0.00 | 22 |
|  | Innsbruck | INN | University Hospital of Innsbruck | 18 | 181 | 1 | 0.01 | 62 |
|  | St. Poelten | STP | University Hospital of St. Pölten | 0 | 4 | 0 | 0.00 | 7 |
|  | Linz | LIN | Kepler University Hospital Linz | 0 | 1 | 0 | 0.00 | 6 |
|  | Salzburg | SAL | University Hospital of Salzburg | 0 | 7 | 0 | 0.00 | 12 |
|  | Vienna | VIE | University Hospital of Vienna - AKH Vienna | 18 | 41 | 1 | 0.02 | 26 |
| France | Amiens | AMI | University Hospital of Amines | 72 | 7 | 1 | 0.14 | 8 |
|  | Angers | ANG | University Hospital of Angers | 91 | 51 | 4 | 0.08 | 18 |
|  | Besancon | BES | Regional and University Hospital of Besancon | 592 | 88 | 24 | 0.27 | 34 |
|  | Bordeaux | BOR | University Hospital of Bordeaux | 140 | 14 | 6 | 0.43 | 24 |
|  | Brest | BRE | University Hospital of Brest | 120 | 14 | 3 | 0.21 | 5 |
|  | Caen | CAE | University Hospital of Caen | 0 | 7 | 0 | 0.00 | 14 |
|  | Clermont-Ferrand | CLE | Regional and University Hospital of Clermont-Ferrand | 0 | 9 | 0 | 0.00 | 19 |
|  | Dijon | DIJ | Dijon University Hospital | 629 | 40 | 16 | 0.40 | 31 |
|  | Grenoble | GRE | University Hospital of Grenoble | 0 | 19 | 0 | 0.00 | 14 |
|  | Lille | LIL | Regional and University Hospital of Lille | 1146 | 18 | 11 | 0.61 | 16 |
|  | Limoges | LIM | University Hospital of Limoges | 0 | 4 | 0 | 0.00 | 4 |
|  | Lyon | LYO | University Hospital of Lion | 188 | 129 | 7 | 0.05 | 36 |
|  | Marseille | MARS | APHM, Public Hospitals Assistance of Marseille- Sainte Marguerite | 0 | 155 | 0 | 0.00 | 49 |
|  | Montpellier | MON | University Hospital of Montpellier | 9 | 24 | 1 | 0.04 | 26 |
|  | Nancy | NAN | University Hospital of Nancy | 0 | 8 | 0 | 0.00 | 9 |
|  | Nantes | NANT | Nantes University Hospital | 701 | 64 | 22 | 0.34 | 16 |
|  | Nice | NIC | University Hospital of Nice | 0 | 1 | 0 | 0.00 | 2 |
|  | Nimes | NIM | University Hospital of Nimes | 152 | 99 | 7 | 0.07 | 34 |
|  | Paris | PAR | APHP, Public Hospitals Assistance of Paris | 97 | 7 | 3 | 0.43 | 15 |
|  | Reims | REI | University Hospital of Reims | 33 | 16 | 4 | 0.25 | 15 |
|  | Rennes | REN | University Hospital of Rennes | 32 | 12 | 2 | 0.17 | 24 |
|  | Rouen | ROU | University Hospital of Rouen | 0 | 18 | 0 | 0.00 | 20 |
|  | Saint-Etienne | SET | University Hospital of Saint-Etienne | 963 | 54 | 7 | 0.13 | 45 |
|  | Strasbourg | STR | Regional and University Hospital of Strasbourg | 0 | 9 | 0 | 0.00 | 11 |
|  | Toulouse | TOU | University Hospital of Toulouse | 0 | 13 | 0 | 0.00 | 7 |
|  | Tours | TOUR | University Hospital of Tours | 261 | 13 | 12 | 0.92 | 18 |
| EnGland | Birmingham | BIR | University Hospitals Birmingham NHS Foundation Trust | 18 | 34 | 2 | 0.06 | 22 |
|  | Bradford | BRA | Bradford Teaching Hospitals NHS Foundation Trust | 0 | 5 | 0 | 0.00 | 10 |
|  | Bristol | BRI | North Bristol NHS Trust | 0 | 6 | 0 | 0.00 | 3 |
|  | Cambridge | CAM | Cambridge University Hospitals NHS Foundation Trust | 0 | 32 | 0 | 0.00 | 31 |
|  | Devon and Exter | DEV | Royal Devon and Exeter NHS Foundation Trust | 0 | 3 | 0 | 0.00 | 9 |
|  | Leeds | LEE | The Leeds Teaching Hospitals NHS Trust | 0 | 1 | 0 | 0.00 | 1 |
|  | Liverpool | LIV | University of Liverpool | 0 | 38 | 0 | 0.00 | 30 |
|  | London 1 | LON1 | Imperial College Healthcare NHS Trust London | 0 | 119 | 0 | 0.00 | 35 |
|  | London 2 | LON2 | University College London Hospitals NHS Foundation Trust | 0 | 21 | 0 | 0.00 | 26 |
|  | London 3 | LON3 | Guy’s and St Thomas’ NHS Foundation Trust | 0 | 5 | 0 | 0.00 | 13 |
|  | London 4 | LON4 | St George’s Healthcare NHS Trust | 0 | 7 | 0 | 0.00 | 3 |
|  | Manchester | MAN | Manchester University NHS Foundation Trust | 12 | 11 | 1 | 0.09 | 16 |
|  | Newcastle upon Tyne | NEW | The Newcastle Upon Tyne Hospitals NHS Foundation Trust | 0 | 2 | 0 | 0.00 | 3 |
|  | Nottingham 1 | NOT1 | Nottingham University Hospitals NHS Trust | 0 | 8 | 0 | 0.00 | 7 |
|  | Nottingham 2 | NOT2 | University of Nottingham, School of lifescience | 0 | 4 | 0 | 0.00 | 28 |
|  | Oxford | OXF | Oxford University Hospitals NHS Trust | 48 | 26 | 0 | 0.00 | 23 |
|  | Plymouth | PLY | Plymouth Hospitals NHS Trust | 29 | 12 | 3 | 0.25 | 23 |
|  | Portsmouth | POR | Portsmouth Hospitals University NHS Trust | 0 | 1 | 0 | 0.00 | 21 |
|  | Southampton | SOU | University Hospital Southampton NHS Foundation Trust | 132 | 2 | 2 | 1.00 | 7 |
|  | Sussex | SUS | Brighton and Sussex Partnership NHS Trust | 204 | 51 | 5 | 0.10 | 32 |
|  | Yorkshire |  | Hull University Teaching Hospitals NHS Trust | 0 | 1 | 0 | 0.00 | 1 |

APHP, Assistance Publique Hôpitaux de Paris; APHM, Assistance Publique Hôpitaux de Marseille; NHS, National Health Service; RWTH, Rheinisch-Westfälische Technische Hochschule; AKH, Allgemeines Krankenhaus; VU, Vrije Universiteit.
